# Educators’ experiences and perspectives of child weight discussions with parents in primary school settings

**DOI:** 10.1186/s12889-022-13210-z

**Published:** 2022-04-22

**Authors:** Nia Coupe, Sarah Peters, Matilda Ayres, Katie Clabon, Alexandra Reilly, Anna Chisholm

**Affiliations:** 1 Lancaster Medical School, Lancaster University, Bailrigg House, Lancaster, LA1 4YE UK; 2 University of Chester, School of Psychology, Parkgate Road, Chester, CH1 4BJ UK; 3 Division of Psychology and Mental Health, Manchester Centre of Health Psychology, Coupland Building 3, University of Manchester, Manchester, M13 9PL UK; 4 Department of Psychology, Bedford Street South, University of Liverpool, Liverpool, L69 7ZA UK

**Keywords:** Childhood obesity, Schools, Communication, Parents

## Abstract

**Background:**

The role of schools in addressing rising childhood obesity levels has been acknowledged, and numerous diet- and physical activity-related interventions exist. Aside from formal interventions, opportunistic parent-educator conversations about child weight can arise, particularly in primary school settings, yet little is known about how useful these are. This study aimed to understand the utility of child weight related conversations with parents through exploring educators’ experiences and perspectives.

**Methods:**

This qualitative study consisted of semi-structured interviews conducted with primary school teaching staff in the United Kingdom (*N* = 23), recruited through purposive and subsequent snowball sampling. Interviews were audio-recorded, transcribed, and analysed using thematic analysis.

**Results:**

Participants identified opportunities and need for child weight discussions in schools. However, conversations were prevented by the indirect and sensitive nature of conversations, and educators’ professional identity beliefs. Using pre-existing face-to-face opportunities, good parent-teacher relationships and holistic approaches to child health and wellbeing were reported as important in optimising these conversations.

**Conclusions:**

Whilst educator-parent child weight discussions are necessary, discussions are highly challenging, with contradictory views on responsibility sometimes resulting in avoidance. Educators’ roles should be clarified, and communication training tailored to increase teacher confidence and skills. Current social distancing will likely reduce opportunistic encounters, highlighting a need to further improve communication routes.

## Background

Childhood obesity is a worldwide epidemic caused by various biological, social, and psychological factors [1, 2]. Latest figures estimate that over a quarter of children in England (2-15 years old) are classed as overweight or obese, and obesity prevalence in 4-5 year olds has risen, from 9.5% in 2017/18 to 9.7% in 2018/19 [3]. Data from Guernsey in the Channel Islands suggest a slightly lower level, with 8.1% of children aged 5-6 classed as obese, though similarly, rates of childhood overweight and obesity have remained high since 2013, with no sign of reducing [4]. Overweight and obesity puts children at higher risk of bullying [5] and is associated with lowered educational attainment [6]. Obesity in childhood also results in being five times more likely to have obesity in adulthood [7] which is associated with increased risk of chronic health conditions like diabetes [8] and cardiovascular disease [9].

Children spend substantial time in schools in most developed parts of the world, making primary schools an ideal setting to intervene. Whilst a wealth of evidence exists for school based dietary and physical activity interventions [10–12], an overview of six Cochrane reviews suggest such multi-component interventions only achieve small reductions in body weight, and findings are limited to high income countries [13]. A meta-synthesis of stakeholder views on the role of schools in obesity prevention highlighted the importance of teachers and parents working in partnership to promote healthy eating and physical activity [14]. However, limited research and guidance exists regarding educators’ (class teachers, head teachers, and teaching assistants) roles in weight management, and if and how such partnerships can be achieved.

Healthcare practitioners have clearer roles in childhood obesity management, for example as part of their child measurement programmes to monitor children’s weight, school nurses calculate the weight status of 4-5 and 10-11 year old children in England [15], and 5-6 and 9-10 year olds in Guernsey (ref). Results are commonly shared with parents via letter, indicating which weight category children fall within. Though the effect of this type of communication is unclear [16, 17], specific guidance exists to facilitate school nurse communication with parents that may occur as a result of these letters [18].

Others recognise that school staff and health practitioners such as school nurses require further skills training in communicating effectively and sensitively with families [19] and that whilst head teachers recognise school is a crucial setting for obesity prevention, they report not having the capability, capacity or confidence to make an impact [20]. A large meta-synthesis of views and experiences of teaching staff, parents and children highlighted that though deemed necessary, these conversations can be sensitive, judgemental, increase stigma, cause negative reactions, and impact children’s self-esteem [21].

Given the frequent contact teaching staff have with children and parents, and the potential for discussions regarding child weight to arise in this setting, it is important to identify if and how such conversations occur. This study therefore aimed to 1) understand current practice in child weight conversations between parents and primary school (ages 4-11) teaching staff and, 2) identify barriers and facilitators to these conversations.

## Methods and materials

This study was a qualitative study comprising of one-to-one semi-structured interviews conducted by co-authored female psychology trainees. A topic guide comprised open-ended questions, and prompts regarding experiences and examples of child weight discussions.

### Participants

Primary school teaching staff (‘educators’) within England (UK) and Guernsey (Channel Islands) were purposively recruited to include eligible individuals with varying age, gender, location, and experience to capture a broad range of views [22, 23]. Teachers were identified through using publicly available contact details on school websites, and invited to take part in telephone interviews via email.

### Procedure

Researchers provided participants with information about the study, which included details about their right to withdraw, procedures in place to ensure confidentiality (research discussed within the research team only, data securely stored) and efforts to anonymise the data (ID numbers replaced participant names, all other names deleted). Following this, all participants provided informed consent to take part. Telephone interviews took place October to December 2019, were audio recorded and transcribed verbatim by research team members. Demographic information was collected concurrently. Whilst ‘data saturation’ is not a goal within a thematic analysis approach given more data collected will likely result in new information [24], the research team felt no new ideas answering the research question were forthcoming. As such, data collection ended once the team agreed sufficient information had been collected to inform the analysis [25].

### Data analysis

Transcripts were analysed using Thematic Analysis [26]. The authors familiarised themselves with the data through reading each transcript twice and making notes to generate initial codes. Codes informed a thematic map which was discussed and compared with initial codes generated by other team members. Transcripts were then coded allowing the team to explore, organise and consolidate the themes through group discussion. Member checking was conducted to enhance trustworthiness of results by sending a summary of results to participants to confirm interpretation and check no pertinent data had been missed [27]. The aim of involving the full team in analysing the data, alongside the member checking, was to minimise bias in the analysis and reporting of the data.

### Reflexivity

We identified some areas for discussion with regards to potential power relations in our work, both within the team, and between researchers and participants [28]. The interviewers were third year students enrolled in an undergraduate psychology degree, and collected the data from participants who ranged in seniority (new teachers through to headteachers). As interviewers were all therefore less senior than interviewees, there may have been less of a perceived power imbalance favouring the interviewer than in other settings. Whilst this power balance may have transferred back to the researchers after the end of the interviews, member checking of the collected data was used to mitigate this.

Secondly, the other three researchers were university staff members, two of which are registered health psychologists and lecturers. This may have left the students feeling less able to disagree with more senior members with regards to interpretation of data. Addressing this, students first analysed the data separately, which was later compared with the analysis conducted by the staff researchers. This addressed both the risks of power imbalances in data analysis, as well as ensured no important concepts were missed from the data.

A further point for consideration is that this school based research was conducted fully by psychologists, who could be viewed as ‘outsiders’ to the world of education, which could have influenced interpretation of the data [29]. Again, member checking may have mitigated against this to some extent. However, researchers can be both insiders and outsiders, for example two of the researchers have school aged children and are familiar with the school environment and have experienced school communication. Nevertheless, these individual experiences may have also influenced ultimate interpretations of the data. A consideration for future research could therefore be including an educator as part of the research team.

### Ethical considerations

As highlighted in the literature, obesity and specifically childhood obesity can be a very sensitive topic and as such can cause distress when raised [19, 21]. Given this study involved school staff only, rather than directly discussing the topic with children and families, we felt there was little risk of distress to participants, however it was made clear to participants before during and after interviews that they were not obligated to participate and could pause or withdraw their participation at any time until analysis of the data. Interviews rather than focus groups were chosen to allow participants to maintain anonymity from their peers, and the interviewers were independent of the schools and not known to participants. During the interviews the researchers adopted a non-judgemental stance to encourage participants to share views and experiences they had that might be perceived as socially unacceptable. These steps were taken to increase the trust of participants and reduce power imbalances.

## Results

### Demographics

Twenty-three educators from 21 schools in England and Guernsey took part in the interviews. Participants comprised of two head teachers, one deputy head teacher (Management- M), one teaching assistant and 19 class teachers (Teaching staff - T). Mean interview length was 21 min (range = 14-33). Index of Multiple Deprivation (IMD) Decile was calculated using postcodes for all English schools using the online tool ([30]) to show the range in the demographics of the school. The IMD decile ranges from 1 (most deprived) to 10 (least deprived), and the range in our data shows a good spread. This information was unavailable for schools in Guernsey. See Table 1 for full demographic information.

**Table 1:** Participant and school demographics

Demographics	
**Participants (*n* = 23)**	
Age range (mean)	*26-62 years (44)*
Gender	*22 female, 1 male*
Years in current school (mean)	*1.5-30 years (9)*
Years in education (mean)	*1.5- 26 years (19)*
**Schools (*n* = 21)**	
Location	
Guernsey	4
Northern England	9
Southern England	4
Midlands	4
**Index of Multiple Deprivation Decile**^a^	
2-4	6
5-7	5
8-10	4

^a^English Schools only (*n* = 15)

### Theme 1. Obesity management discussion triggers and gaps

**Fig. 1:**
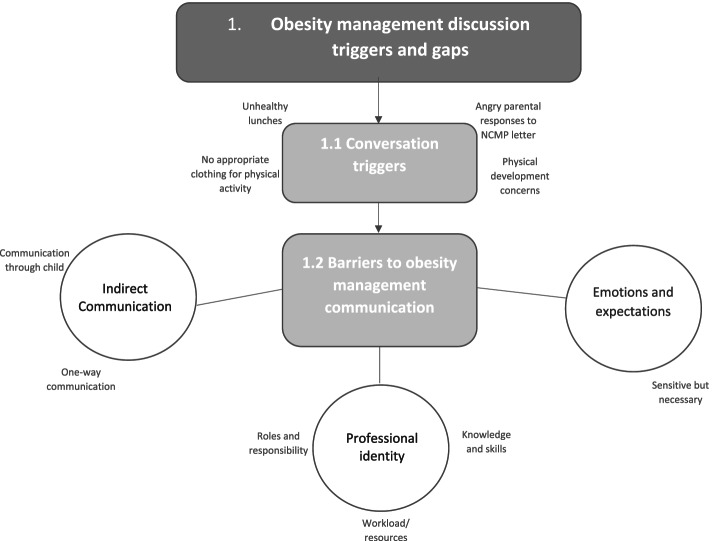
Thematic Map of Obesity Management Opportunities and Barriers in Primary Schools

This theme addresses the aim regarding understanding current practice in schools. It incorporates the need for educator-parent conversations given common issues arising around children’s diet, physical activity and development. Despite the highlighted need for these conversations, this theme also includes a range of barriers that prevent these conversations from taking place. An overview of these themes can be seen in Fig. 1, and are discussed in more detail below.

### Conversation triggers

Educators discussed four common issues that prompted weight-related discussions with parents. Firstly, educators highlighted that in contrast to the healthy lunches provided by schools, packed lunches provided by some parents were unhealthy. Educators described examples of extremely unhealthy lunches (e.g. *“a pack of Rolos and a Mars bar”* (T7)) and identified these unhealthy lunches as triggers for educator-parent conversations. as

*“[unhealthy packed lunches] the thing that you notice with children who are overweight”* (T2).

Secondly, educators highlighted that some children regularly forgot their physical education (PE) kits (e.g. suitable clothing for physical activity lessons), either to avoid participation in physical activity due to self-consciousness, or *“because nobody provides them with it”* (T13). Whilst some schools reported that *“not having a PE kit doesn’t mean that you don’t do PE”* (T20), other schools had stricter policies in place: *“…as policy stands if you haven’t got your kit then you’re not doing it”* (T6). The lack of suitable clothing to enable children to participate in school based physical activities was therefore a trigger for conversations with parents.

The third trigger described by educators was that weight-related conversations could occur as a result of concern over the impact of a child’s weight on their psychological, social, physical and educational development/attainment.

*"Very often it’s not just about their weight but it affects everything about them, their behaviour, their attitudes to learning overall, their ability to form friendships, everything about them is impacted because of low self-esteem".* (T19)

The fourth trigger was the NCMP letters sent to parents about their children’s weight. Educators explained although they were not involved in the NCMP, they had to deal with parental responses to the “*hard hitting letter(s)”* (M11). This included directly managing parental complaints and managing parent distress caused by receiving the letter, despite teachers not being privy to the NCMP data themselves.

*"We have experienced parents being upset from those results but then they aren’t directly linked to the school’s data." (T18)*

### Barriers to regular educator-parent communication

Despite the identified need and opportunities for child weight conversations with parents, educators also reported that conversations weren’t happening regularly.

#### Indirect communication

A barrier identified across the dataset was that despite opportunities, *“teachers don’t speak directly to parents about children’s weight at all”* (T14). Few reasons were identified for this, discussed below.

##### One-way communication

Messages regarding for example healthy lunchbox policies were mostly communicated through letters, email and apps, and as such meant there was little opportunity for two-way conversations.

*"Parental letters at the beginning of each term state far more clearly this is what we are expecting you to put in the packed lunch." (T6)*

Teachers were not always aware of the information being sent home to parents because “*a lot of what gets sent home is done by email and I don’t get all the emails.” (T18).*

##### “Drip feeding” education through children

Whilst educators stressed that children were well educated around healthy living through the curriculum, this information was not necessarily *“getting past the kids to the parents”* (T20). This was important in relation to behaviour change, given “*parents are buying and feeding them*” (T5).

Though educators echoed this feeling that educating the parents through the children did not have much impact, this approach was also viewed as *“a good way through to parents, even the most stubborn ones” (T19)*

#### Emotions and expectations

Weight and obesity were described as very sensitive topics*, “because it is such a personal thing”* (T6), making conversations “*awkward” (T7, M11, T15, T16), “tricky” (T2, T4, M11, T22) and “difficult” (T6, T7, T9, T15, T20)* for parents and teachers alike.

Educators expressed that broaching this topic with parents could be perceived as *“attacking their parenting*” (T15) and being “*frightened of offending people”* (T10) was a barrier to conversations taking place. This meant these conversations were something that *“most teachers would prefer to avoid*” (T2). However, educators felt that it shouldn’t be a taboo topic because it was so important to child health.

*"It’s not comfortable, it is never a comfortable conversation, but you know it is one that has to be had." (T13)*

#### Professional identity

##### Conflicting roles and responsibilities

There were opposing views among educators about their role with parents. Staff managers felt a responsibility to speak with and educate parents and that *“schools have got a great opportunity because we can communicate with the parents really easily”* (M12). Teaching staff were divided with some adamant that bringing up the topic was “*not my place*” (T10), expressing that it was not within their job remit:

*"[Parents] wouldn’t like to be told that their child is overweight, and I don’t actually think that is my job. I’m supposed to be there as an educator." (T20)*

Others felt rightly or wrongly, the responsibility “*kind of falls on our shoulders*” (T13) but also felt that *“the balance is too far towards schools having to be expected to do that” (M12).*

Educators also expressed that they were restricted by school or wider policy, meaning that they *“aren’t allowed to discuss child weight specifically with parents”* (T4), with one headteacher going so far as to say that bringing up weight with parents was illegal:

*"We are not, legally, we are not allowed to directly approach parents and tell them". (M1)*

##### Knowledge & skills gaps

Educators highlighted that as they did not have the appropriate *“medical backgrounds”* (T2), for managing these conversations, and that they’d “*rather a medical professional to be responsible for it” (T13)* because they are *“better suited to doing that” (M12).*

Similarly, others felt that they didn’t have the skills or knowledge required to start and manage these conversations successfully and thought they “*almost need educating as teachers on how to deal with that” (T7).* Teachers were also unclear as to who they should discuss or refer such problems, with this topic also not having been included in staff training.

*"It is swept under the carpet a little bit, I’ll be honest with you. I’m trying to think if there’s been an inset day or has there been any training or has it ever been mentioned, I’m not sure if it’s even in any of our policies about what you do with a child who’s obese, I really don’t know and that’s bad really." (T8)*

##### Competing workload and resources

Educators expressed that they felt restricted by resources, and without additional capacity to address child weight with parents.

*"Our workload is already completely huge and pretty unmanageable… All we can do is what we already do, educate them on healthy foods, exercise and how to stay fit and healthy*.*"* (T18)

A suggested solution to this was to work in partnership with health services to meet these needs., albeit “*quite a long way off from achieving that at the minute” (M12).*However, this was also resource-restricted, with school nurse funding and availability having *“been withdrawn and so there is no school nurse” (T19).*

### Theme 2. Factors affecting conversation outcomes

**Fig. 2:**
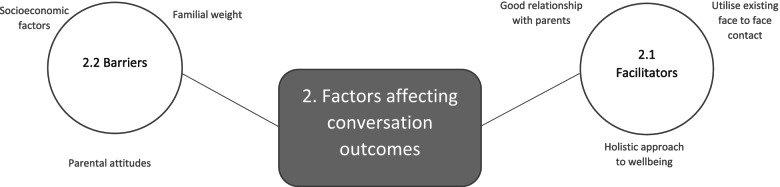
Thematic Map of Factors Affecting Conversation Outcomes

Despite identified missed opportunities, educators recalled examples when they had discussed a child’s weight or related behaviour with the parents, which highlighted some important factors that positively and negatively affected conversation outcomes. These are depicted in Fig. 2 and discussed below.

### Successful conversation facilitators

#### Establishing relationships

Educators saw an already established relationship with parents as crucial to parents changing health-related behaviour as a result of conversations, expressing that having “*no background knowledge of a child or the family…would be very negative*.” (M11). In particular, this would reduce message receptiveness and ability to tailor conversations appropriately.

“*If you don’t have a relationship with the parent, they won’t listen to you… it’s all family specific*.” (T20)

#### Holistic approach

Educators perceived a holistic approach to conversations as more effective, preferring to focus conversations on children’s health as opposed to their weight specifically. For example, one positive strategy reported was to focus on something a child was good at to encourage participation in extracurricular physical activity.

*"Picking on something that they’re really good at and so the parent thinks that you’re encouraging them because they’re good at something rather than because they’re overweight. That works very well actually."* (M1)

Focusing on the curriculum and expected development levels of a child was also identified as a good starting point as it made it less difficult for educators to initiate the topic and start a conversation.

*"I was able to say the physical development of the children is a priority in school and in the early years because it’s a prime area in the early curriculum so being able to say that, gave me the lead really to talk about it [weight]."* (T9)

#### Use existing points of contact

Educators highlighted that using existing *“non-threatening situations*” (M1) like parents’ evenings were a good opportunity to discuss issues around weight and related behaviours as parents may feel less singled out or stigmatised.

*"Doing it at parent’s evening is quite successful because they will assume that you are talking to all the parents about it… If you directed straight to them, then they can get quite defensive, cos they’ll be aware that their child is overweight*.*"* (M1)

Parents evenings were also identified as good opportunities to educate parents around healthy eating, as they could reach all parents as opposed to only reaching those already interested in healthy eating in invited parent workshops, which was described as *“Kind of like preaching to the converted”* (T16).

### Barriers to successful outcomes

#### Socioeconomic factors

Socioeconomic status was highlighted as a factor accounting for the range in observed child weight-related behaviours in school, with educators noting that diet tended to be poorer in families living in more deprived areas.

*"*W*hereas previously I’ve taught in sort more deprived areas and there is a real difference… it was definitely something we’d notice in terms of packed lunches and yeah, all that sorts of thing." (T22)*

Enforcing rules on food brought into school was identified as tricky because it potentially placed *“more pressure on a family that could be already struggling” (T21),* particularly given the reflection by M11 that they were *“directing more and more of our parents to foodbanks”.*

Cost was also a barrier to some parents supporting children to take part in extracurricular physical activity, particularly those who needed it:

*"I have a little boy, he was amazing at tennis… I spoke to the tennis coach actually, and said ‘look finances will be really difficult’ ‘oh don’t worry I’m sure we can come up with something.’….but he didn’t, he never went to a tennis lesson." (T20)*

However, promoting physical activity outside of school did not solely rely on financial resources, because it was also an issue in privately educated families where sedentary behaviour was due to “*real issues with [online] gaming” (T3).*

#### Parental and familial weight

Parents’ and family members’ weight was viewed to confer additional difficulty to child weight conversations. For example, by reducing parent engagement and recognition of obesity-related risk, or need to act on that risk.

*"I think the key phrase that’s always stuck in my head is ‘but we’re a fat family’. And they were …overweight, sort of grossly obese …and they just didn’t see that [weight management programme] as something that they really wanted to engage with." (M12)*

Furthermore, family background and ethnicity meant that body weight perceptions differed between families and was sometimes experienced as a barrier to successful conversations:

*"Some of our cultures of children that we are working with, it is a sign of wealth if you are overweight …so there’s also that cultural element that we’re pushing against". (T16)*

#### Parental attitudes to healthy eating policies

Some conversations around lunchbox contents were reported to result in parents “*adapt[ing] lunches accordingly*” (T16) suggesting some success. However, educators reported struggling to get parents on side, where conversations “*taken quite badly*” (M11), or resulted in *“very little change” (T20).*

When these issues were tackled by changing school policy, educators said that parents *“rebel against that” (T2)* as they don’t like to be told what to feed their children.

*"The parents were up in arms with ‘how dare you tell us how to feed our children at lunch time!"* (T15)

Where healthy eating policies were in place, educators felt that *“parents will interpret things as they want to”* (T4) for example including “*raisins covered in yogurt coating*” as a fruit portion (T4), or simply not adhering to rules.

*"They’re not allowed chocolates or biscuits in there, I mean they sneak them in but they’re not supposed to." (P10)*

Educators reported that some parents insisted that their children *“won’t drink water*” (T15) or “*don’t eat fruit” (T4),* making conversations challenging, and policies hard to enforce.

### Overview of findings and current practice

**Fig. 3:**
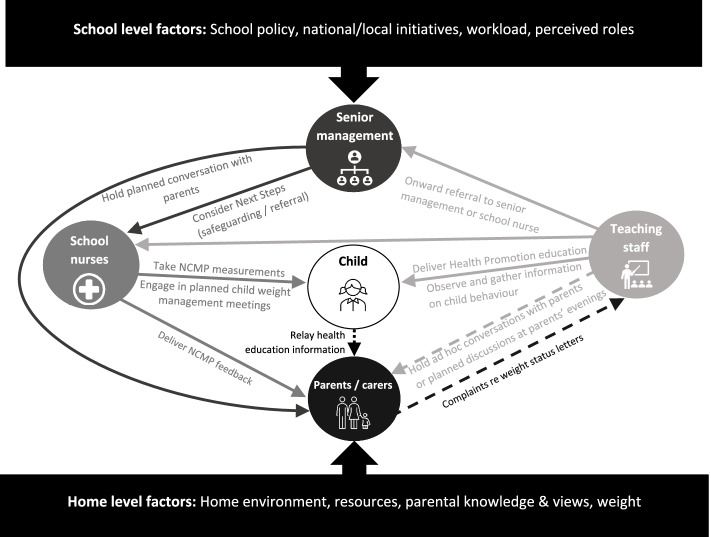
Overview of Primary/Elementary School Obesity Management Communication and Influencing Factors

Taken together, these two core themes highlight how educators reported obesity management discussion to occur within schools, and how contextual factors intervene in such discussions. Figure 3 displays a visual representation of the school-based communication (verbal/ written) pathways identified in the data, as well as the contextual factors at both home- and school-level that influence the perceived success of discussions that were held.

## Discussion

The results depict a picture of current obesity management communication within primary schools from educators’ point of view. This highlights that despite common triggers for such conversations, communication is restricted by: existing approach to communication, school and wider policy, resources, professional identity, concerns over sensitivity of topic and potential to offend, and the exclusion of teachers from the NCMP. Teachers on the whole avoided direct communication with parents but tended to focus on educating children around healthy living in line with the curriculum, and where necessary referred children to school nurses or senior leadership team members. Where sporadic direct discussion, or opportunistic indirect discussion occurred, educators felt that communication was most effective when a good relationship with parents existed, and when existing face-to face contacts such as parents’ evenings were utilised to raise weight-related issues as part of a more holistic approach.

Though indirect communication through children was identified as a barrier to parent communication, reinforcing these healthy living messages to children is highlighted by Ofsted as part of an important part of a school’s role [31]. However, others have highlighted that children’s understanding doesn’t translate into behaviour [32], supporting our findings that more direct routes to educating parents may be required.

Ofsted [31] highlight in their report, that school lunchboxes were probably not contributing to the obesity crisis, yet this was perceived as problematic by teachers in our study and one of the main triggers for weight-related conversations with parents, particularly in relation to parents not adhering to school policy. Our findings support previous research that both recognised parents as part of the solution to childhood obesity, and identified a lack of parental support by schools in supporting healthy eating [14]. Recent work by Goldthorpe and colleagues [33] exploring the implementation of a healthy school initiative suggests that school policies were more likely to be accepted by parents when they had a shared sense of responsibility and perception of lifestyle norms with school staff. Though this also supports our findings regarding parental attitudes, it is unclear how changing these could be achieved more practically. In particular, people living in more deprived communities where obesity rates are higher have further barriers to living healthily in relation to access and availability of suitable foods and leisure facilities, as well as limited financial resources for these [34]. Though free school meals can address this to some extent, these barriers continue outside of school hours, where issues around holiday hunger and food insecurity have been further exacerbated by the Covid-19 pandemic [35] Building partnerships between parents and school staff is one important aspect to a multi-faceted approach to childhood obesity, but will not work alone in the absence of additional support and resources for families to implement healthier lifestyles.

Within our data, not involving teachers in the NCMP was a missed opportunity to engage more constructively with parents in these weight-related conversations, particularly given school nurses are largely no longer based within UK schools. Addressing this would require further evidence regarding availability of school nurses and where they are best placed, in order to inform policy. This is likely to be impacted by the COVID-19 pandemic, given school staff and nurses may have additional responsibilities and less time to dedicate to weight-related concerns.

Goldthorpe et al. [33] concluded that effective two-way teacher-parent communication was vital for successful implementation of their intervention, suggesting the one-way communication identified across the schools in our study may be an important factor to improve to optimise future school obesity management communication. Similar results have been reported in Swedish settings, where improving parent-teacher communication and cooperation was identified as important to ensure changes are made at home, specifically in low SES groups [36]. This is important given that SES was a barrier to behaviour change in our study, and supports previous findings that further resources and governmental support are key to improving schools’ roles in obesity management, particularly in more deprived areas [37].

### Limitations

Though some commonalities will exist, our results may be specific to schools based in England and Guernsey. Further work is required to confirm if the same issues exist within and outside of these areas, where the child measurement programmes do not exist and infrastructure differs.

Data collection took place prior to the COVID-19 pandemic, meaning some important factors may have presented themselves since. Social distancing policies in schools may have resulted in fewer opportunities for informal teacher-parent discussions. However, efforts by schools to improve communication in the likelihood of home schooling (such as increased use of email and apps) may present further communication opportunities, though these are likely to be education focused.

## Conclusions

This study contributes to the limited evidence base by providing a clearer understanding of current obesity management communication in UK primary schools, and identifies ways to increase and optimise these opportunities. To reduce missed obesity management opportunities and maximise those that occur, schools should include training that clearly outlines roles, responsibilities, and procedures for teaching staff responding to child weight-related issues, and clear routes for two-way communication should be available and used effectively. Effectiveness of conversations may also improve through building relationships with parents. External skills training may be required to ensure communication is effective and increase teacher confidence.

## Data Availability

The datasets used and/or analysed during the current study are available from the corresponding author on reasonable request.

## Abbreviations

IMD: Index of Multiple Deprivation
NCMP: National Child Measurement Programme
PE: Physical Exercise
